# Supplementary material for: Deterministic formation of carbon-functionalized quantum emitters in hexagonal boron nitride
Source: Nat Commun. 2025 Dec 13;16:11450. doi: 10.1038/s41467-025-66314-6 (PMC12749744; doi:10.1038/s41467-025-66314-6)
Supplement: Supplementary file 1 — Supplementary Information [file 41467_2025_66314_MOESM1_ESM.pdf]

Supplementary Information

Deterministic formation of carbon-functionalized quantum emitters in hexagonal boron nitride

Manlin Luo[1†], Junyu Ge[2†], Pengru Huang[3†], Yi Yu[1], In Cheol Seo[1,4], Kunze Lu[1,5], Hao Sun[3], Jian Kwang Tan[1], Beng Kang Tay[1,6], Sejeong Kim[7], Weibo Gao[1,5], Hong Li[2,6*], Donguk Nam[8*]

[1]School of Electrical and Electronic Engineering, Nanyang Technological University, Singapore, Singapore.
[2]School of Mechanical and Aerospace Engineering, Nanyang Technological University, Singapore, Singapore.
[3]Institute for Functional Intelligent Materials (I-FIM), National University of Singapore, Singapore, Singapore.
[4]Quantum Innovation Centre (Q.InC) & National Metrology Centre (NMC), Agency for Science, Technology and Research (A*STAR), Singapore, Singapore.
[5]Division of Physics and Applied Physics, School of Physical and Mathematical Sciences, Nanyang Technological University, Singapore, Singapore.
[6]CINTRA CNRS/NTU/THALES, IRL 3288, Research Techno Plaza, Nanyang Technological University, Singapore.
[7]Department of Electrical and Computer Engineering, Sungkyunkwan University (SKKU), Suwon 16419, Republic of Korea.
[8]Department of Mechanical Engineering, Korea Advanced Institute of Science and Technology (KAIST), Daejeon, Republic of Korea.

[†]These authors contributed equally to this work.
[*]Corresponding authors: dwnam@kaist.ac.kr; ehongli@ntu.edu.sg

Supplementary Note 1: Tip sample preparation

Chromium (Cr) tips are fabricated by employing polystyrene (PS) spheres as a mask. These PS spheres were obtained from YUAN BIOTECH, with diameters ranging from 20 nm to 1 mm. For this study, a PS sphere diameter of 3.5 μm was chosen, resulting in a tip pitch of approximately 2 μm, for a proper resolution during measurements.

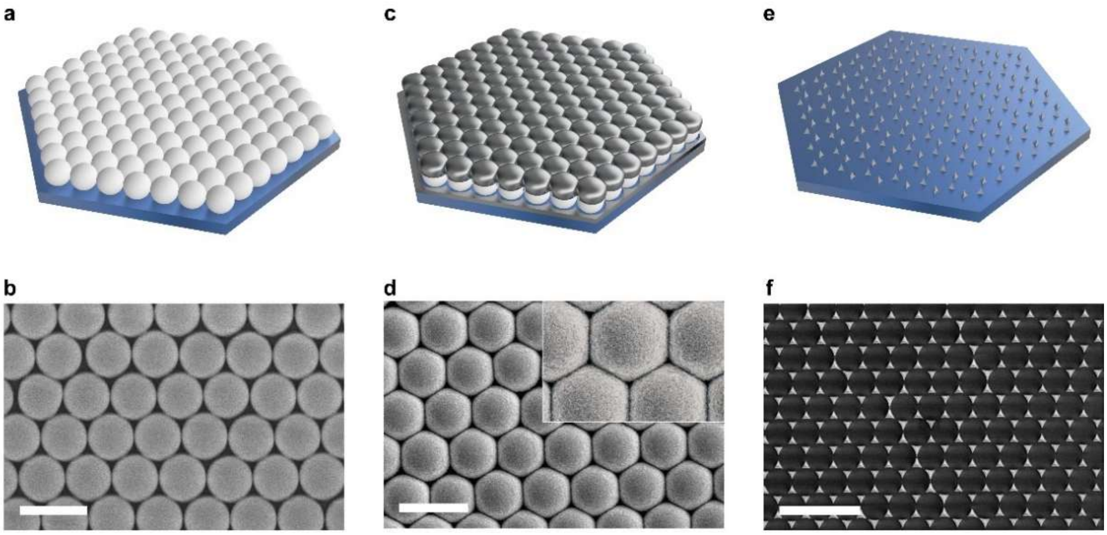

Supplementary Figure S1 | Schematics and scanning electron microscope (SEM) images of the formation of chromium (Cr) tips. a–b, PS spheres are spin-coated and distributed uniformly as a monolayer on the Si substrate. Scale bar: 5 µm. **c–d,** Cr is evaporated onto the PS spheres for 400 nm. Scale bar: 5 µm. Inset: a zoom-in photo of (b), showing a shrinking gap between spheres. Scale bar: 2 µm. **e–f** Cr tip array after removing PC spheres. Scale bar: 10 µm.

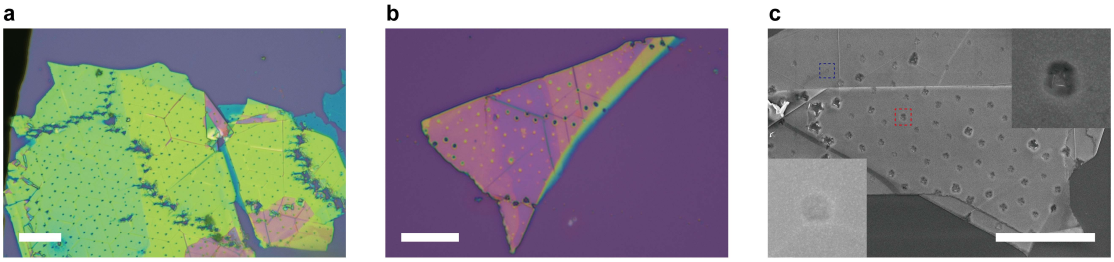

Supplementary Figure S2 | Photos of indented hBN. a–b, Optical microscope images and **c,** SEM photos of the indented hBN flakes. The inset in c shows two indented nanoindentations with different depth. The scale bars are 10 μm.

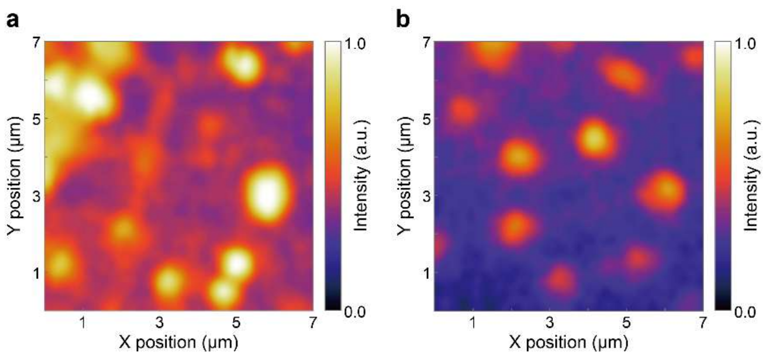

Supplementary Figure S3 | Confocal PL mapping of the same sample before and after annealing. a, PL map acquired after nanoindentation and carbon insertion, but before annealing. Strong background emission and bright spots likely arise from excess carbon residues on the hBN surface. **b,** PL map of the same region after annealing at 850°C in argon. Nanoindentation sites become clearly identifiable with significantly reduced background noise. Both maps are normalized to the same intensity scale for direct comparison.

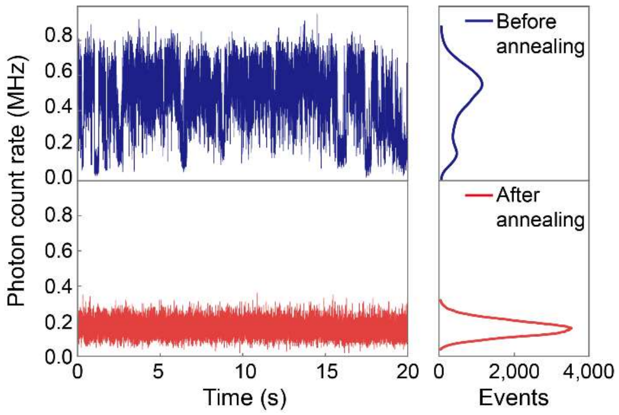

Supplementary Figure S4. Real-time SPAD count monitoring of a selected emitter before and after annealing. Time-resolved single-photon count rates were recorded at the same emitter location before (top, blue) and after (bottom, red) annealing. Before annealing, the signal was highly unstable with pronounced fluctuations and intermittent blinking behavior. After annealing, the same site demonstrated a significantly reduced count rate (~0.2 MHz), but the emission became considerably stable over the entire measurement window. The histograms on the right display the corresponding distributions of count events over the same time window. Before annealing, the broad and skewed distribution reflects strong temporal fluctuations, while after annealing, the distribution narrows considerably, indicating improved temporal stability and reduced blinking.

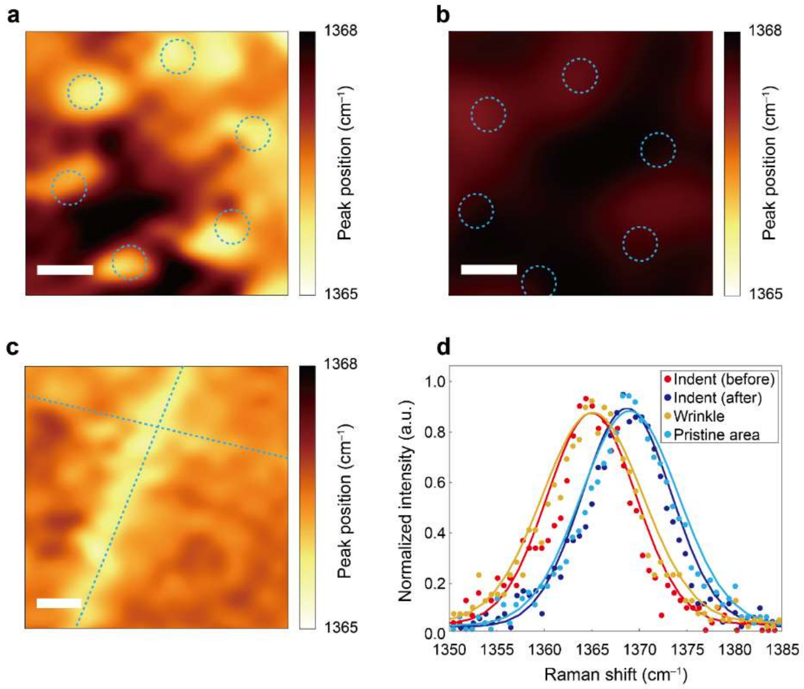

Supplementary Figure S5 | Raman characterization of strain in hBN. a, Raman 2D map of peak positions of bare-tip-indented hBN before annealing. Dashed circles indicate nanoindentation positions. **b,** Corresponding Raman 2D map after annealing, acquired at the same location. The strain of the hBN peak at nanoindentations before annealing was clearly released after annealing. **c,** Raman map of an hBN flake with wrinkles after annealing. Dashed lines highlight the wrinkle features. The wrinkle retains its red shift. **d,** Normalized Raman spectra extracted from the regions indicated in a–c: nanoindentation sites before annealing (red), after annealing (dark blue), wrinkle (yellow) and pristine area (light blue). All measurements were performed on the same sample under identical experimental conditions. Scale bars: 1 μm.

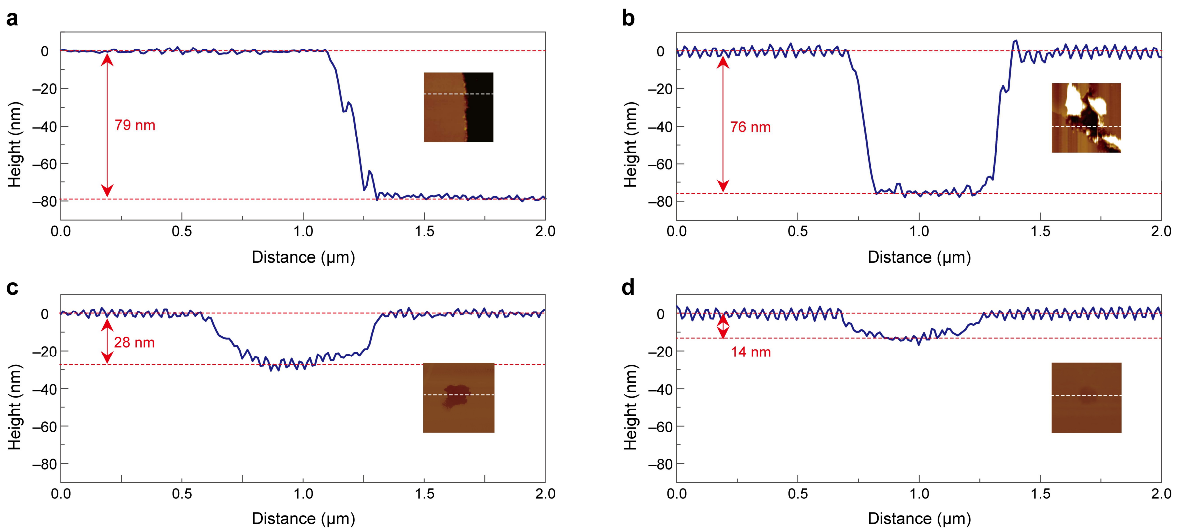

Supplementary Figure S6 | Atomic force microscopy (AFM) profiles of an indented hBN sample. a, Line profile at the edge of the hBN flake, indicating a flake thickness of 79 nm. **b–d,** Line profiles taken over three nanoindentations with different depths.

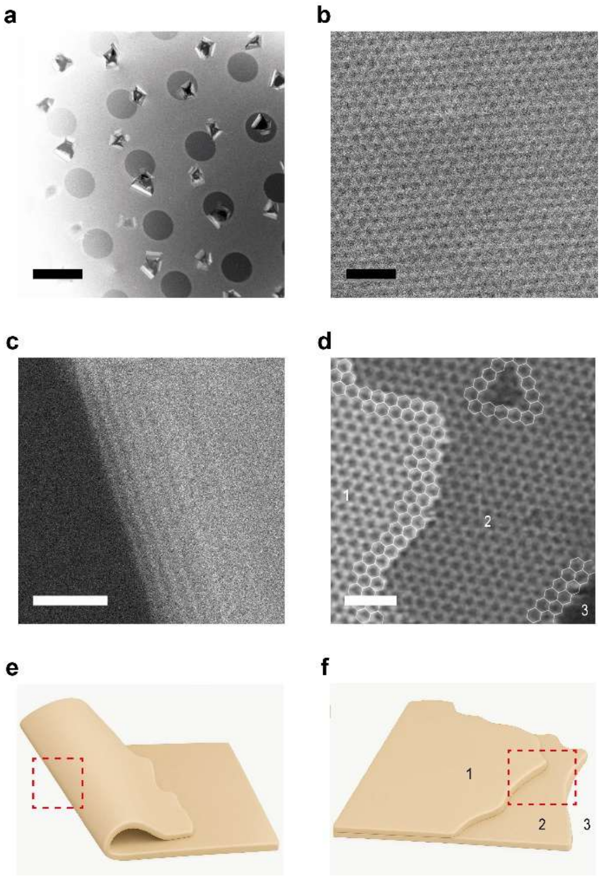

Supplementary Figure S7 | AC-TEM images of indented hBN. a, Low-magnification TEM image of indented hBN transferred onto a Quantifoil TEM grid. Scale bar: 2 µm. **b–d,** HAADF-STEM images of (b) a pristine hBN region, (c) a folded edge and (d) a fractured edge region. **e–f,** Schematic illustrations corresponding to panels (c) and (d), respectively, highlighting (e) the multilayer folded edge and (f) the fractured edge. Red dashed boxes mark the regions shown in (c) and (d). Labelled areas indicate: 1–multilayer region; 2–monolayer region; 3–open hole region. Scale bars: (a) 2 µm, (b,d)1 nm, (c) 2 nm.

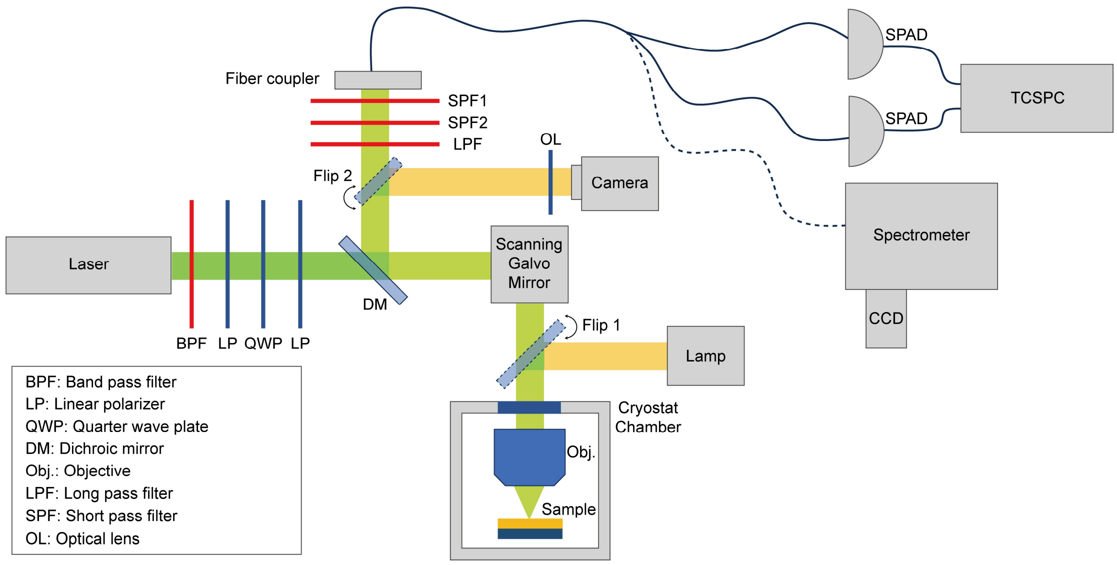

Supplementary Figure S8. Schematic illustration of the experimental set-up.

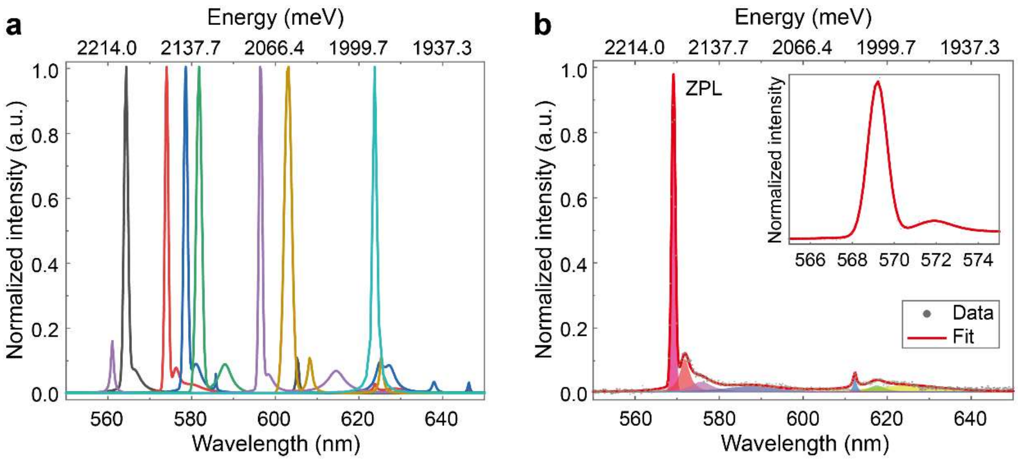

Supplementary Figure S9 | Representative normalized photoluminescence (PL) spectra. a, Photoluminescence spectra taken from various nanoindentation sites in one carbon-inserted hBN flake, showing diverse emission wavelengths and narrow full width at half-maximum (FWHM). **b**, Photoluminescence spectrum of a selected emitter featuring a FWHM less than 1 nm. Grey dots represent measured data, the red solid line represents the fitting. Inset: Close-up of the zero-phonon line (ZPL).

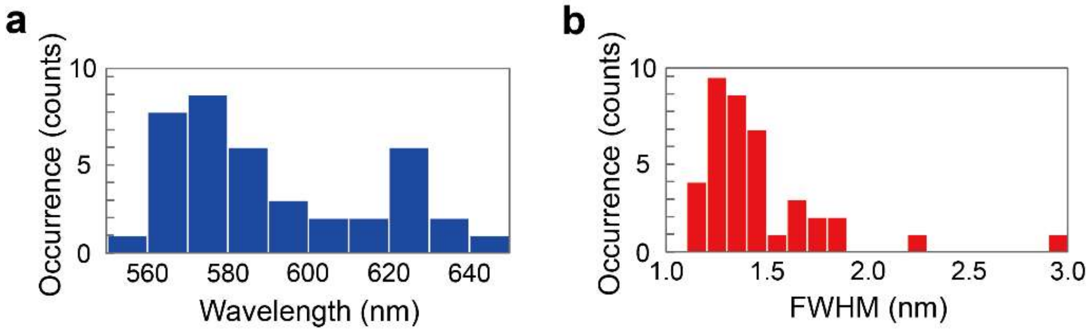

Supplementary Figure S10 | Statistical study of 40 SPEs. a, ZPL distribution. b, FWHM distribution.

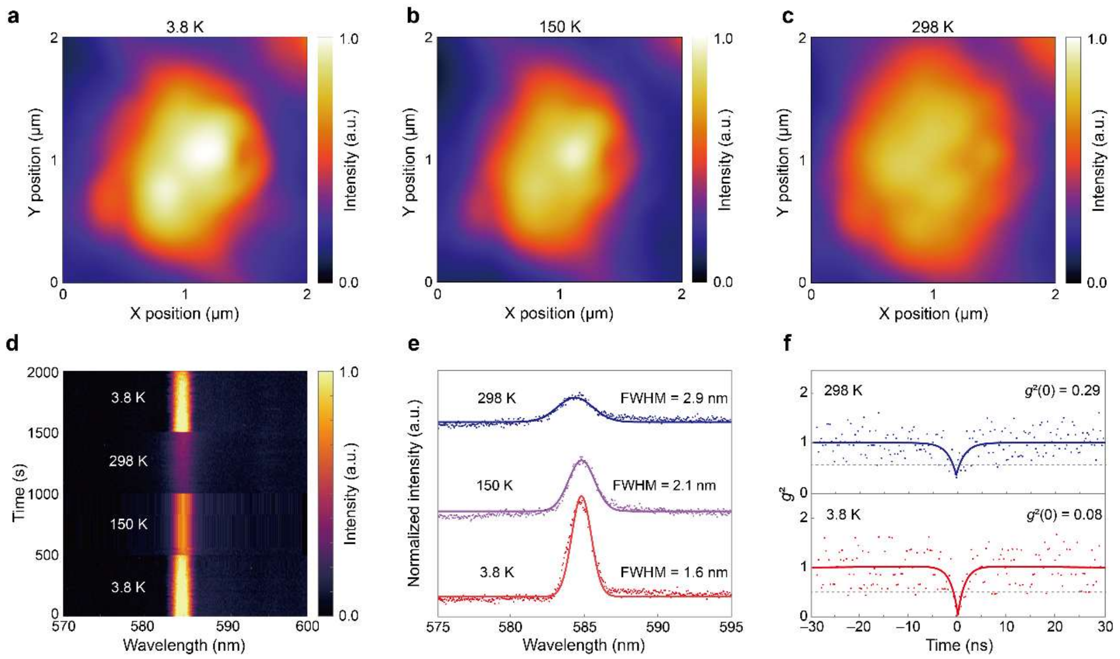

Supplementary Figure S11 | Temperature-dependent characterization of a SPE a–c, Normalized PL intensity maps measured at (a) 3.8 K (low vacuum), (b) 150 K in low vacuum, and (c) 298 K in ambient air. All maps were normalized using the same intensity scale to enable direct comparison. **d,** Time-resolved PL spectroscopy of the same emitter recorded sequentially under 3.8 K, 150 K, 298 K, and back to 3.8 K. **e,** Normalized PL spectra extracted under each temperature condition. **f,** Autocorrelation function of the same SPE measured at 3.8 K (low vacuum, bottom) and room temperature (ambient air, top).

Supplementary Note 2: PL real count rate calculations

The real count rate can be calculated based on the measured count rate as below:

$$CR_{real} = (CR_{meas} \times CF - DC) \times QE \times TE$$

$$= (CR_{meas} \times \frac{1}{1-C \quad _{meas} \times T_{dead-time}} - DC) \times QE \times TE$$

CR_{real} is the real count rate, CR_{meas} is the measured count rate, CF is correction factor, DC is the dark count rate of the detector, QE is the detector quantum efficiency, TE is the coefficient of the optical path transmission.

In our case, the $T_{dead-tim}$ is 77 ns, the DC is ~50 cps, the QE of the detector is ~49%, and the TE is 4.62. The coefficients of the optical path transmission are calculated by multiplying the transmission rate of optical components on the optical path.

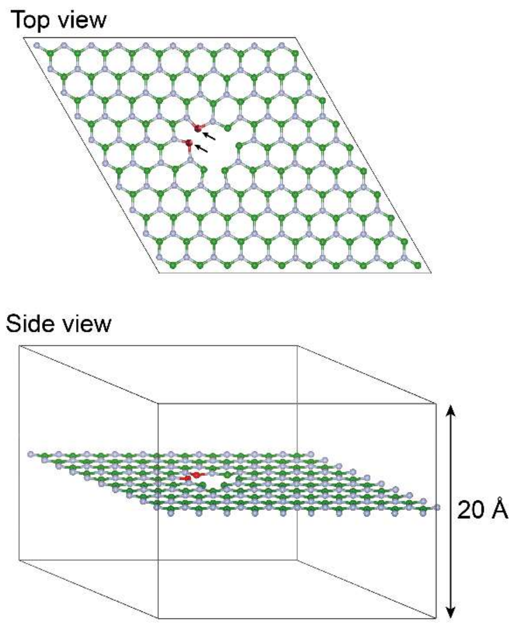

Supplementary Figure S12 | Top and side views of the monolayer hBN supercell model used in DFT calculations. A supercell containing a monolayer of hBN with an inserted carbon defect is shown (atom code: boron: green; nitrogen: grey; carbon: red).

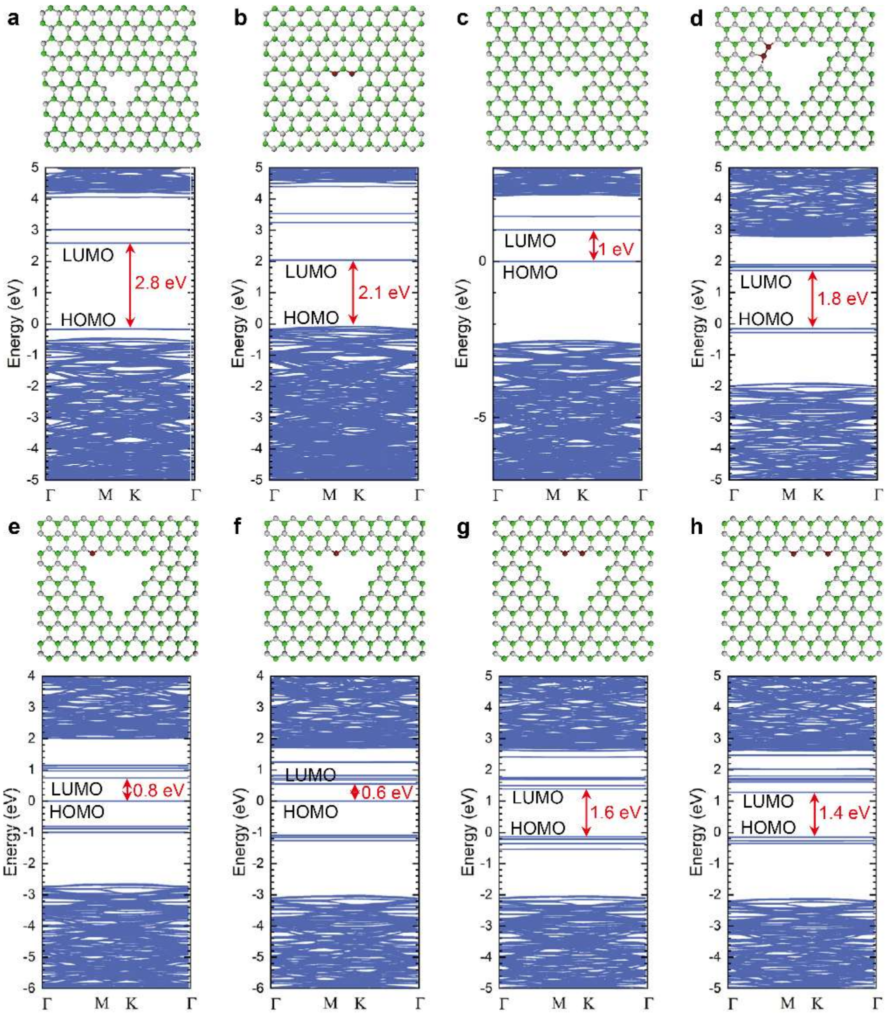

Supplementary Figure S13. Representative atomic structures and corresponding calculated electronic band structures (color coding: B in green; N in grey; C in red).

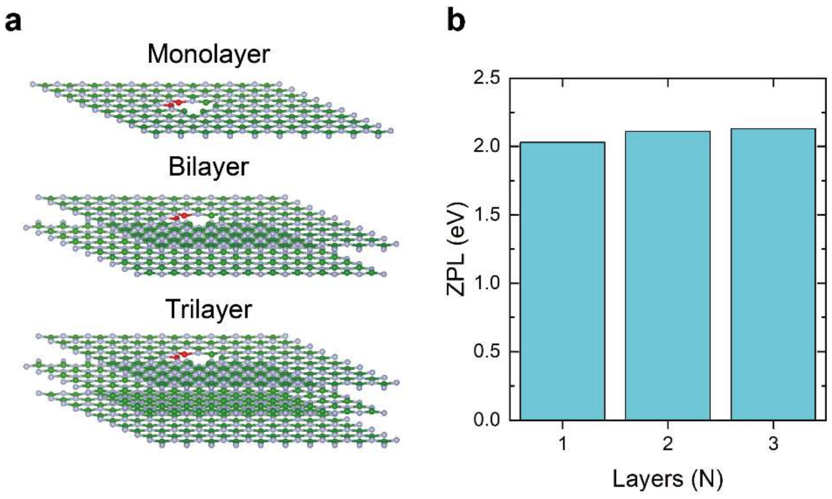

Supplementary Figure S14 | DFT-calculated HOMO–LUMO gaps of carbon dimer defects in hBN. a, Atomic structures of the monolayer, bilayer, and tri-layer models. **b,** Calculated HOMO–LUMO gaps of the carbon-dimer defect structures in the monolayer, bilayer, and tri-layer models.
